# Wound monitoring of pH and oxygen in patients after radiation therapy

**DOI:** 10.1186/s13014-019-1413-y

**Published:** 2019-11-11

**Authors:** Steffen Auerswald, Stephan Schreml, Robert Meier, Alexandra Blancke Soares, Maximilian Niyazi, Sebastian Marschner, Claus Belka, Martin Canis, Frank Haubner

**Affiliations:** 1 Department of Otorhinolaryngology, University Medical Center Regensburg, Regensburg, Germany; 2 Department of Dermatology, University Medical Center Regensburg, Regensburg, Germany; 3 Presens, Biopark, Regensburg, Germany; 4 Department of Otorhinolaryngology, University Medical Center Munich, Klinikum Großhadern, Marchioninistr. 15, 81377 Munich, Germany; 5 Department of Radiation Oncology, University Medical Center Munich, Munich, Germany; 6 German Cancer Consortium (DKTK), German Cancer Research Center (DKFZ), Heidelberg, Germany

**Keywords:** Wound healing, Radiotherapy, Luminescence, pH, Oxygen

## Abstract

**Objectives:**

Postradiogenic wound healing disorders are an important clinical problem. While a variety of treatment modalities are available, there is no strategy to objectively judge treatment success. The aim of this study was to evaluate a 2D luminescence imaging system for pH and oxygen in non-healing wounds after radiotherapy.

**Methods:**

Luminescence 2D imaging was performed with the VisiSens (Presens, Regensburg, Germany) 2D imaging systems A1 and A2 for oxygen and pH, respectively. Biocompatible planar luminescent sensor foils were applied to non-irradiated and irradiated skin as well as to radiogenic wounds of five patients and the pH and the oxygen saturation was determined.

**Results:**

pH measurements showed significant differences between non-irradiated skin (6.46 ± 0.18) and irradiated skin (6.96 ± 0.26). Radiogenic wounds exhibited the highest pH values (7.53 ± 0.26). Oxygen measurements revealed a mean oxygen saturation of non-irradiated skin of 6.19 ± 0.83 mmHg. The highest value of oxygen saturation (28.4 ± 2.4 mmHg) was found on irradiated skin while irradiated wounds had a poor oxygen saturation (9.4 ± 2.2 mmHg) (mean ± s.e.m.).

**Conclusion:**

We found that routine measurement of pH and pO2 in patients could be easily integrated into the clinical routine. The results of the measurements show unfavorable pH and oxygen saturation conditions for wound healing in irradiated wounds. Interestingly, irradiated wounds exhibit a more pronounced hypoxia than irradiated skin which is reflected by an altered pH and pO2 compared to unirradiated skin, which has the potential to serve as a prognostic marker in the future. In addition to the objectification of the treatment success of postradiogenic wound healing disorders, the extent of skin toxicity could already be predicted during radiotherapy with this method.

## Introduction

Squamous cell carcinoma continues to be the most common of the head and neck malignant tumors. Depending on the progress of the disease, surgery as well as primary radiotherapy with or without concomitant chemotherapy have become established as treatment modalities. Salvage surgery is used for recurrences, but re-irradiation is also considered on a case-by-case basis [1, 2]. However, it is known that radiotherapy leads to short and long-term changes in the irradiated tissue and is therefore associated with an increased risk of wound healing disorders [3–5]. Complications include infection [6], skin atrophy, tissue fibrosis [7], ulcerations, fistula formation [8] and in rare cases vascular rupture. The healing rate after flap transplantation also appears to be reduced [9]. A review by Lee, Chin et al. reports a rate of flap losses of 9.8% [10], while other studies report wound healing disorders after free flap reconstruction in irradiated areas in up to 40% of cases [11].

The management of these complications requires extensive efforts [12] and is even further complicated by a lack of assessment criteria to objectively measure treatment success. Thus, the current follow-up strategy for these complications is the independent observation and subjective assessment of the wounds by several physicians.

Previous studies on chronic wounds have shown that adequate tissue oxygenation and pH regulation are essential for cell proliferation and protein synthesis [13, 14]. Moreover, cell turnover, migration and enzymatic activity are affected by changes in pH und subsequent activation of various proton-sensing G protein-coupled receptors (GPCRs) [15].

The processes underlying wound healing are dormant in intact skin and are activated to restore the barrier integrity upon injury. This healing processes consist of three phases, inflammation, proliferation and remodeling [16, 17]. If the wound healing is functional, the pH of the wound decreases steadily, from pH values in the alkaline range (around pH 8–8.5) to the pH of intact skin upon completion of the healing process (5.5–6) [18]. However, if the pH remains at a high level, wound healing slows down or stops, partially by reduced cell proliferation and migration as seen in chronic wounds [13, 19]. In addition, chronic wounds exhibit a centripetal increase in pH, which is due to a centrifugally upregulated sodium / hydrogen exchanger isoform 1 (NHE1) expression [20]. This leads to an increased hydrogen-ion extrusion and thus acidification of the wound in the periphery [21, 22]. The low pH was shown to inhibit keratinocyte migration, proliferation and viability. Similarly, microvascular endothelial cells exhibit increased adhesion and lower migration rates in low pH conditions. In addition, there is a reduction in the release of mediators such as IL-6 and IL-8 [23, 24] and interferon-γ from keratinocytes in the acidic wound periphery compared to the wound center. The reduced IL-6 and IL-8 secretion potentially inhibits keratinocyte recruitment from the wound periphery to the center. Interferon-γ stimulates VEGF production, which is a key molecule for neoangiogenesis, implying that this process could be inhibited by the low pH in the wound periphery [25]. In conclusion, a low pH in the periphery of the wound seems to inhibit processes that are essential for functional wound healing. Similarly, the oxygen partial pressure (pO_2_) differs in acute versus chronic wounds, with a pO_2_ of about 60 mmHg in acute wounds without epithelialization and [26] an average of 35 mmHg in chronic wounds. Based on these findings, a monitoring of the pH and pO_2_ suitable for everyday clinical practice could facilitate the objective assessment of wound healing disorders so that wound management could be adapted and improved. The aim of this study was to collect first in vivo data in irradiated tissues of patients with wound healing disorders after primary or adjuvant radiochemotherapy. To determine these parameters, a newly developed camera system was used allowing the direct measurement on a cell layer.

## Materials and methods

### Study subjects

All volunteers (*n* = 5, AVG = 66 y, 51-77y, male = 5) suffered from wound healing disorders in previously irradiated areas. These wound healing complications developed under irradiation (*n* = 4) or emerged after functional surgical procedures (*n* = 1, Patient 1, Fig. 4) in previously irradiated tissues. The measurements were carried out between June 2016 and February 2018 at the University Medical Center Regensburg, Germany. At the time of measurement, the radiation had been finished for 23 months on average. A detailed list of patients can be found in Fig. 3.

### pH/o2 imaging

Luminescence 2D imaging was performed with the VisiSens 2D imaging systems A1 and A2 (PreSens, Regensburg Germany) for oxygen and pH, respectively. The technique uses biocompatible planar luminescent sensor foils (SF-HP5R for pH, SF-RPSu4 for O_2_, PreSens GmbH, Regensburg, Germany) for 2D read out of the respective parameters.

The sensor foils consist each of an analyte permeable sensitive sensor layer on a transparent support foil. The O2 sensitive sensor foil consists of a red luminescent indicator dye that is incorporated in an oxygen permeable polymer layer. Oxygen can diffuse into the layer and can interact with the indicator dye. The fluorescence of the indicator dye gets quenched, when an oxygen molecule collides with it, which is called the effect of collisional quenching. Thereby the red indicator fluorescence decreases with increasing oxygen levels. A second inert reference fluorophore is incorporated in the sensitive layer, which provides a stable green reference signal. The ratio of red and green signals results in a referenced 2D O2 image [27]. The pH sensor foil on the other hand incorporates a green fluorescent pH indicator dye that displays bright green fluorescence behavior in its protonated state at lower pH levels, whereas the green fluorescence gets weaker in deprotonated state at higher pH levels. The green pH sensitive fluorescence shows a typical sigmoidal response behavior. The signal is referenced on a second red fluorescence dye in the sensitive layer that results in a pH-insensitive inert signal. Ratio of red and green hereby results in a referenced pH response.

**Fig. 1:**
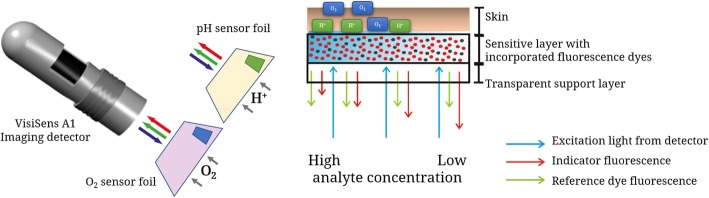
Schematic drawing of the pH and pO2 measurement system including a USB-handheld camera

The detector unit is a small handheld USB-powered camera system with incorporated excitation LEDs and fluorescence filters. The excitation LEDs shine filtered blue light at the respective sensor foil and excite the luminophores that are enclosed in the sensitive layer of foil. The optical filters separate excitation and emission light. The sensor foils emit red and green signals (indicator signal and reference signal, respectively), which are collected in the wavelength separated red and green channel of the RGB camera detector and stored in a RGB color image. The RGB referencing scheme used here is described in detail in previous works [28] (Fig. 1).

The resulting RGB image is then split into the separate color channels and a ratio (R) image of the red and green information is calculated. This ratio R can be correlated to the respective analyte value via a mathematical calibration function. The data obtained in the 2D images can be further used for analysis. Data recording and evaluation is done in the VisiSens Analytical Software (PreSens, Regensburg, Germany) provided with the systems.

For the experiments, pH and O2 sensor foils of a size of 2 × 2 cm were used. The O2 sensor foils (SF-RPSu4) were applied untreated. pH sensor foils (SF-HP5R) need to be soaked with aqueous solution (Ringer solution, Braun, Germany) for at least 30 min prior to use to ensure fast and sufficient proton exchange between sensor and sample.

The pH sensors were calibrated using at least 6 different phosphate buffers [in the range from 5,3 to 8,04 [29]. The buffers were filled into the wells of a calibration helper plate (CP-HP5R, PreSens GmbH) that contains the sensors from the same batch. For the O_2_ sensor foils a two-point calibration in oxygen-free environment (aqueous sodium sulfite solution) and air saturated environment (ambient air) was performed. The imaging software was used to calibrate the sensor signals and compute the quantitative maps from the raw sensor response images. In the analyte maps, a region of interest was defined over the respective area of interest (non-irradiated skin, irradiated skin, wound). After using the time series to ensure a steady state, the mean O2 and pH values could be determined.

### Measurements

All measurements took place during hospitalization, surgery or outpatient treatment. The measurements were integrated into clinical routine with a duration of about 15 min per measurement. The time required for preparation and measurement was documented. A photo documentation of the wound conditions was performed.

**Fig. 2:**
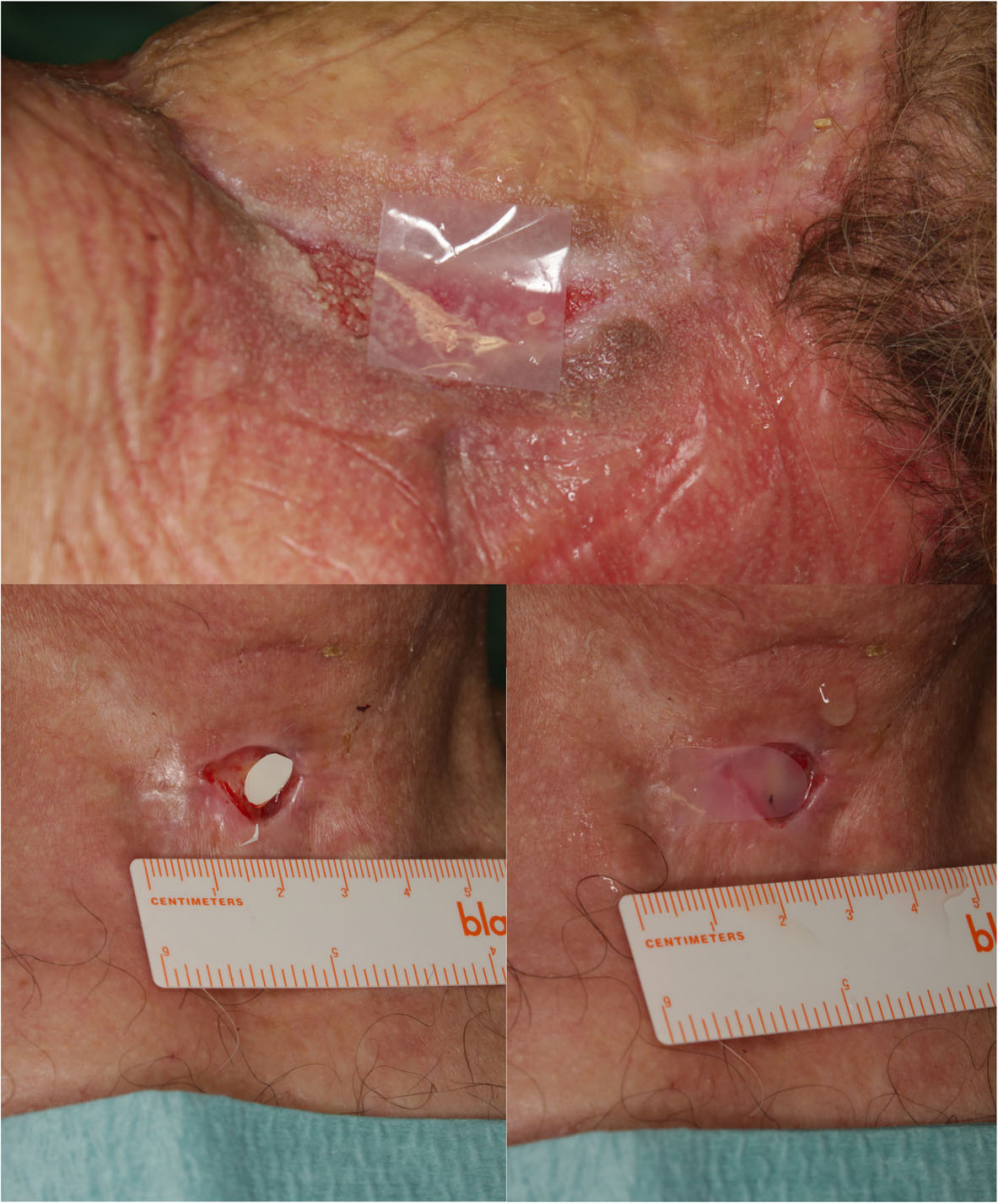
Clinical examples of the sensor foil application in wounds of different locations

The prepared sensor foils were carefully applied to the ROIs (1. the unirradiated skin on the arm or back. 2. the wound base without intact skin. 3. The irradiated skin next to the wound base). For smaller wounds or irregular surfaces, the sensor foils’ shape and size were adjusted with Wullstein scissors (213,314, Karl Storz, Germany). After making sure that there is no air between the film and the surface, at least 2 min were allowed to develop a steady state between the sensor and sample surface before measurement (Fig. 2). Subsequently, a consecutive series of images (ten recordings, time interval: 2 s) was acquired and analyzed as described above.

### Statistics

After the ROIs have been defined the mean values were calculated in each area, resulting in 6 values per subject (3 measurements each for O_2_ and pH). The values were analyzed for statistical significance using the one way analysis of variance (ANOVA) comparing non-irradiated skin, irradiated skin and wound surface) We used Sigma Plot 13.0 (Systat Software Inc., Chicago, IL, USA) and SPSS (IBM Corp., Armonk, NY, USA) for all analyses.

*p* < 0.05 (*) was considered to be significant and *p* ≤ 0.001 (**) to be highly significant. Data are given as mean and standard error of the mean (s.e.m.).

### Statement of ethics

Informed written consent was obtained prior to the study. The experiments were conducted in accordance with the sixth revision (Seoul, Korea, 2008) of the Declaration of Helsinki (1964) and the local ethics committee gave approval (No. 06/171: 2007, University of Regensburg, Germany).

## Results

### Patients

**Fig. 3:**
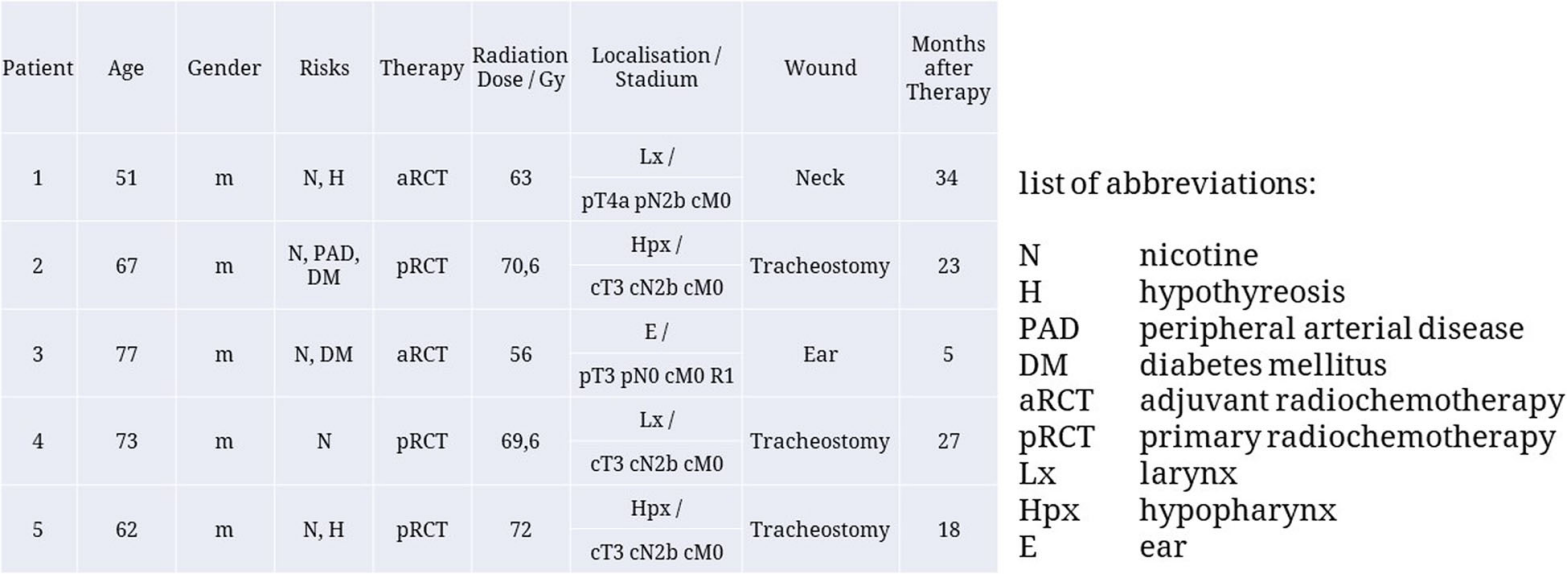
Clinical characteristics of patients included into the study

This study included 5 male patients between the ages of 51 and 77 who presented with wound healing disorders after adjuvant or primary radiochemotherapy (Fig. 3).

### Application in clinical routine

For each patient, senor foils were applied to three regions of interest (ROIs) and measured with the VisiSens 2D Imaging System. Depending on the experience of the examiner, each measurement could be taken in 4 (+/− 1) minutes. The pH sensor foil preparation as well as the setup of USB-microscope and laptop took another 10 min and were performed by a nurse. The O2 sensor did not require any preparation. The measurements of O2 and pH were performed separately (Fig. 2).

**Fig. 4:**
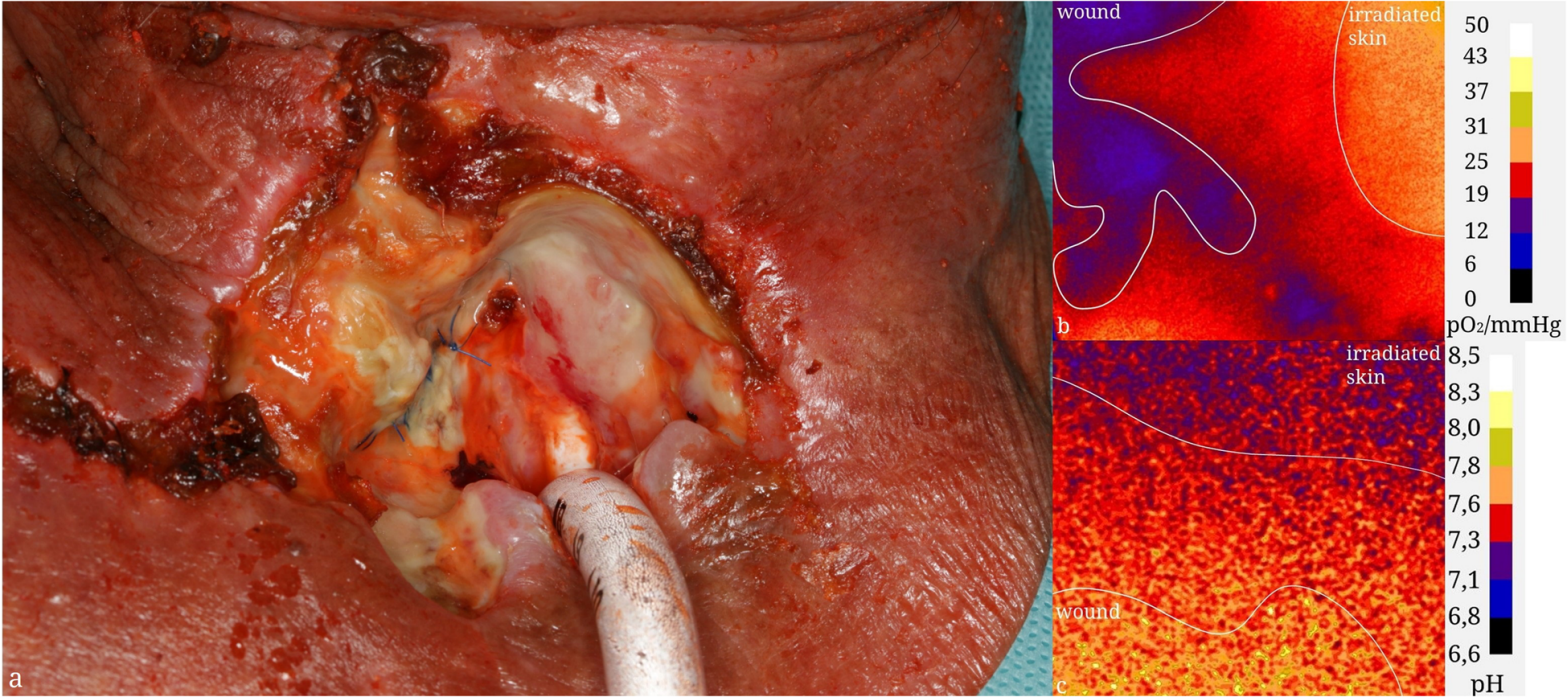
Clinical Case of a 51-year-old male, three years after laryngectomy and radiochemotherapy who suffered from severe wound healing disorder after treatment of a voice fistula insufficency. **a** photo documentation. **b** pO2 sensor response. **c**) pH sensor response

**Fig. 5:**
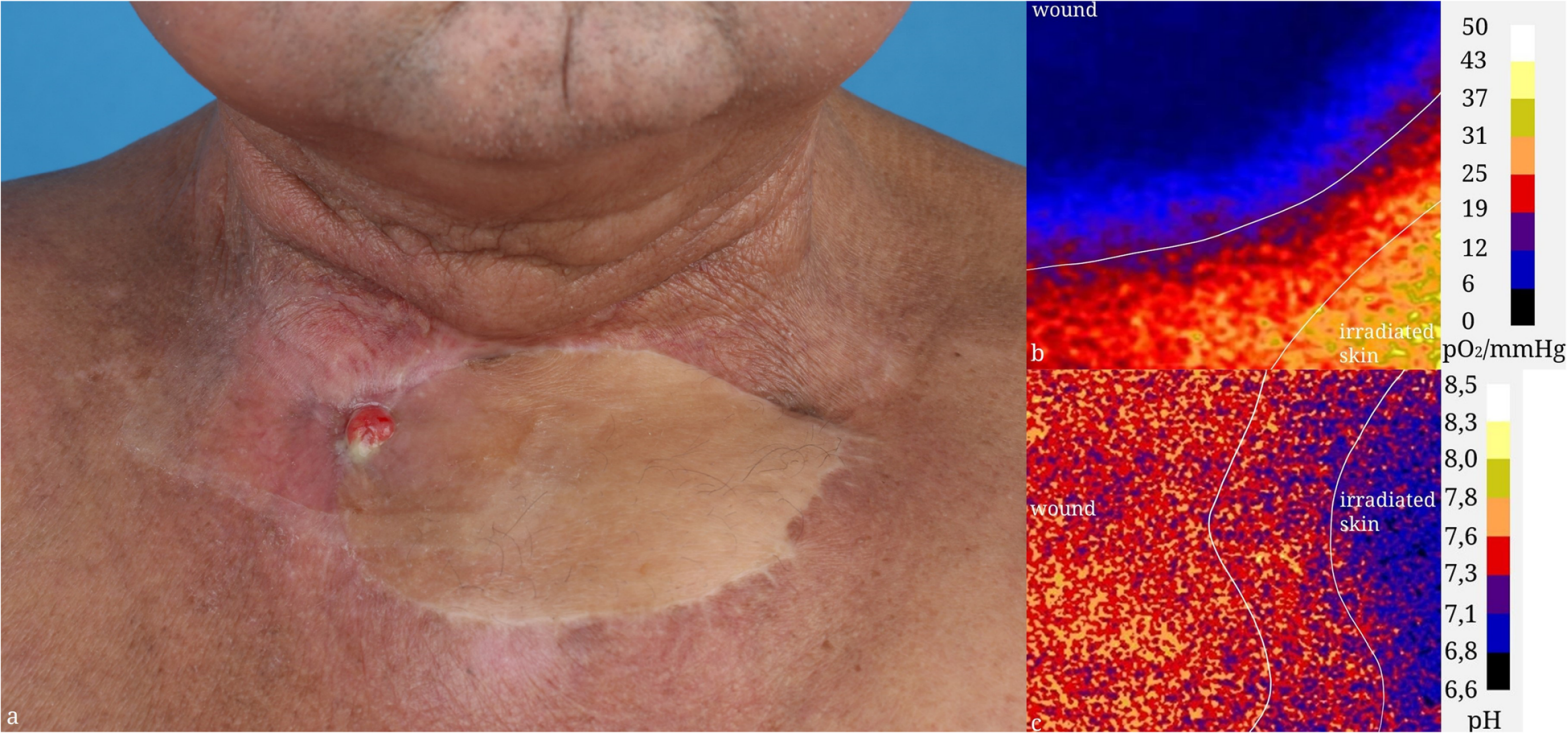
Clinical Case of a 62-year-old male, suffering from a wound healing disorder after closure of a tracheostomy and an infection of the sternoclavicular joint two years after primary radiochemotherapy of a cT3 squamous cell carcinoma of the hypopharynx. **a** photo documentation. **b** pO2 sensor response. **c**) pH sensor response

Figures 4 and 5 show a photo documentation of clinical cases as well as the false color display of pH and O2 saturation of chronic wounds.

### pH – measurements in chronic wounds, irradiated skin and unirradiated skin

**Fig. 6:**
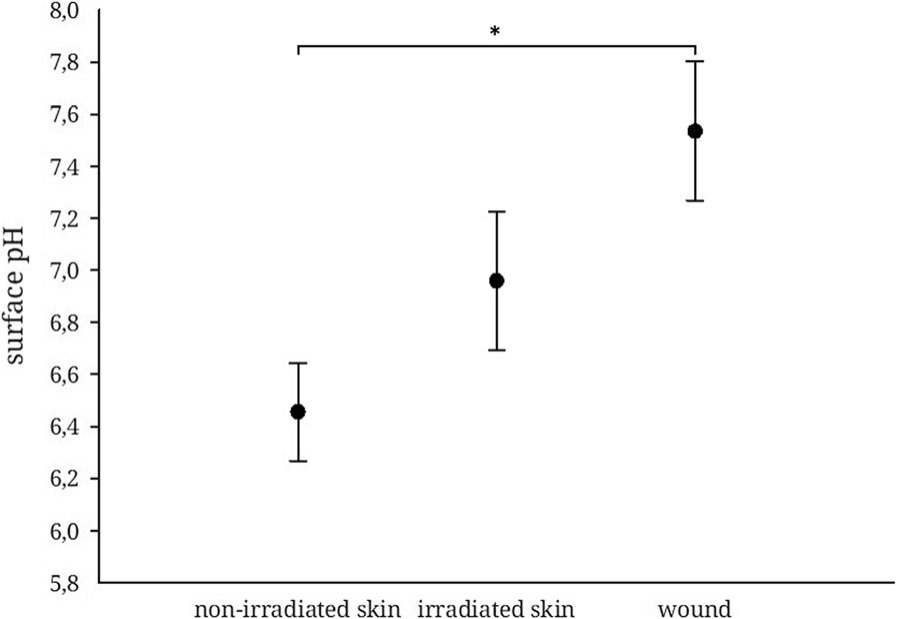
pH values in non-irradiated skin, irradiated skin and irradiated wound. (*n* = 5, mean ± s.e.m., *p* ≤ 0,05, * *p* ≤ 0,05, ANOVA)

pH values were determined by defining a representative region of interest on the quantitative map of pH values from the area of interest (non-irradiated skin, irradiated skin, wound) and calculating the mean pH of this region. The non-irradiated skin on the arm or back of the patient had a pH of 6.46 ± 0,18 (mean ± s.e.m.). The pH of the irradiated skin next to the wound base was 6.96 ± 0,26 (mean ± s.e.m.). The irradiated wound presented with a pH of 7.53 ± 0,26 (mean ± s.e.m.), which is significantly higher than the pH of non-irradiated skin. There was no statistical significance between the other groups (Fig. 6).

### O_2_ – measurements in chronic wounds, irradiated skin and unirradiated skin

Similar to the pH measurements, pO_2_ values were determined by defining a region of interest on the quantitative maps of pO_2_ values from the different areas of interest.

**Fig. 7:**
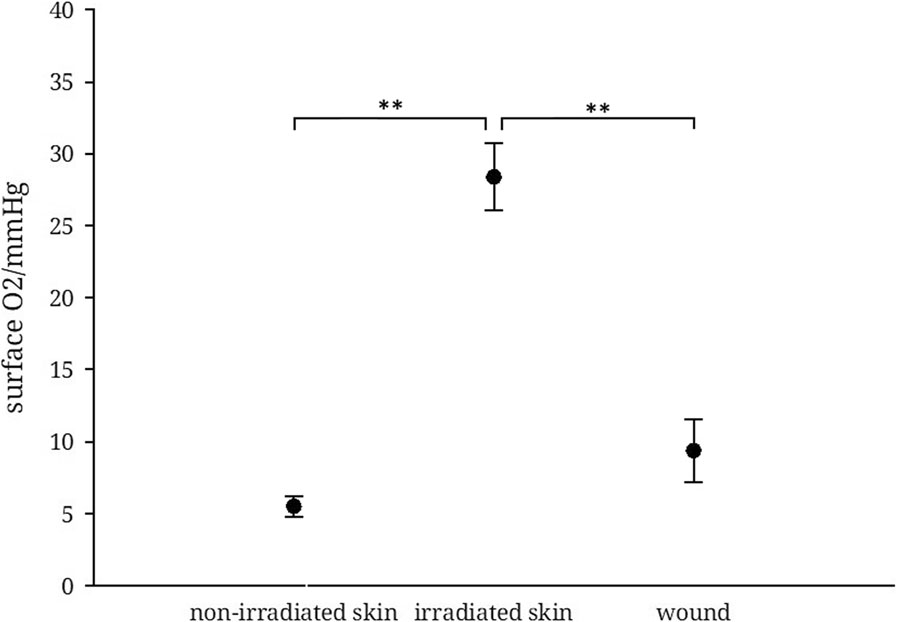
0_2−_ saturation in non-irradiated skin, irradiated skin and irradiated wound. (*n* = 5, mean ± s.e.m., *p* ≤ 0,001, ** *p* ≤ 0,001, ANOVA)

The mean oxygen saturation of unirradiated skin was 6,19 ± 0,83 (mean ± s.e.m.) mmHg, implying an intact barrier function. In contrast, irradiated skin had a highly significantly higher oxygen saturation with 28,4 ± 2,4 (mean ± s.e.m.) mmHg, while the oxygen saturation in the irradiated wound was 9,4 ± 2,2 (mean ± s.e.m.) mmHg, highly significantly lower than in irradiated skin. There was no statistically significant difference between the results in non-irradiated skin areas versus irradiated wound (Fig. 7).

## Discussion

Wound healing complications can be side effects of radiation therapy [12].To address wound healing disorders in clinical practice, several approaches are pursued. An important progress was the intensity modulated radiotherapy (IMRT) by careful adaptation of the radiation field which should improve the therapeutic benefit and reduce side effects [30]. Computer-assisted inverse planning of IMRT and application with modern high-precision accelerators enables optimal sparing of surrounding normal tissues [31, 32]. As radiation techniques get better and treatment fields get smaller, re-irradation might occur more often resulting in a potential higher number of patients with wound healing disorders in previously irradiated areas. The resulting toxicity may lead to further wound healing disorders.

Various therapeutic approaches to manage wound healing disorders have already found their way into clinical routine and here complement the conventional wound management. Objective measurements analyzing the wound bed and the effects of intervention are still missing. A clinical review of our group summarizes the current treatment strategies in wound healing complications after radiotherapy [12], including hyperbaric oxygen, hydrogel membranes [33] and pluripotent stem cells [34].

Wound healing disorders in otorhinolaryngology are sometimes sustained not only by the tissue damage resulting from the previous irradiation. In the case of pharyngocutaneous fistula, the additional contamination by saliva and the associated bacterial colonization also has a negative effect on the treatment [6]. In recent years, negative-pressure wound therapy has established as an efficient treatment method [35–37]. The wound is separated from the environment by means of a pressure-tight dressing. Using a portable pump negative pressure is built up to − 200 mmHg continuously or intermittently [38]. As a result, the wound base is cleared of secretions, granulation tissue is built up, thus promoting wound healing. Numerous clinical studies have now indicated that thus reducing the treatment time and a better outcome can be achieved.

In addition to the methods mentioned in the management of postradiogenic wound healing disorders, numerous others are discussed [12]. Common to all, however, is the problem of objectively assessing treatment success. Schreml et al. studied the pH [26] and oxygen [14] of intact skin as well as in acute and chronic wounds in detail. In addition, they established a method of 2D visualization of these parameters [13]. We adopted this method to develop a better understanding of the conditions in postradiogenic wound healing disorders.

While his group describe a pHe of about 6.5 in the periphery of the wound, we found values of 7.53 ± 0.26 (mean ± s.e.m.) in our experiments. Taking into account the molecular biological relationships described above, the conditions for wound closure in postradiogenic wounds appear to be more favorable with regard to the environment at the wound edge. However, pH-gradients play a crucial role in recruiting keratinocytes inwards from the wound edges.

Significantly less favorable are the conditions with regard to the oxygenation of the wound base in irradiated wounds. The oxygen partial pressure in acute wounds without epithelialization is about 60 mmHg [26] while chronic wounds have an average of 35 mmHg. A centripetal distribution pattern as in the case of the pH value is not found here [13]. While the oxygen barrier is regenerated with re-epithelialization, the measured values usually fall continuously [26]. Our measurements showed very pronounced hypoxia at 9.4 ± 2.2 (mean ± s.e.m.) mmHg and without any epidermal barrier. Since sufficient tissue oxygenation is crucial for keratinocyte proliferation and migration in healing [14, 39], extremely unfavorable conditions seem to exist for efficient wound healing. Should these results be confirmed in studies with larger numbers of cases, hyperbaric oxygen therapy as a therapeutic approach could address this problem. As described above, it is increasingly being used in the treatment of osteoradionecrosis. Thus, by improving the oxygen partial pressure, it could prove to be an effective therapy for wound healing disorders after radiotherapy in HNSCC patients. A Cochrane analysis by Bennett et al. in 2016 [40] found only two studies from 1999, in which hyperbaric oxygen therapy, applied pre- and postoperatively, described a significant advantage in the case of head-neck salvage surgery. However, these studies are only of limited significance due to methodological deficits.

The cause of the extremely hypoxic conditions in irradiated non-healing wounds could be damage to small vessels and fibrosis due to the radiation [41]. However, this will have to be investigated in future works.

On unirradiated skin we found a pH of 6.46 ± 0.18 (mean ± s.e.m.) and an oxygen saturation of 6.19 ± 0.83 (mean ± s.e.m.) mmHg. Low oxygen saturation on intact skin has already been described with this measurement method [26]. Due to the intact stratum corneum, the sensor foil is continuously deprived of oxygen without being refilled, which results in a reading of approximately 0 mmHg. Striking, however, is the high pH compared to previous work [42]. Using a similar measurement method, the authors described pH levels of about 5.1 in a patient population of a similar age. However, in contrast to our study, the skin areas on which the measurements were performed were standardized in that no cleaning or local application of care products was carried out 24 h before the pH was measured. Furtjes et al. describe an increase in pH after soap and water cleaning of the skin to up to 6.47 [43]. Our pH measurements were made in everyday clinical practice, so standardization in unirradiated skin was not part of our protocol. Thus, the difference in pH measured in this study compared to the aforementioned study could be due to personal hygiene or disinfecting measures.

In this study, we have for the first time measured the pH and pO_2_ on irradiated skin. Here we found an average pH of 6.96 ± 0.26 (mean ± s.e.m.) and an oxygen saturation of 28.4 ± 2.4 (mean ± s.e.m.) mmHg. Within the stratum corneum, the formation of free fatty acids [44], the production of urocanic acid [45] as well as the expression of NHE1 [20] among others are responsible for the acidic pH of human skin. Deeper layers of the stratum corneum have a more alkaline pH, reaching pH neutral levels [18, 46, 47]. Together with the observation that measured oxygen levels on intact skin are close to 0 mmHg [26], the results indicate that the skin surface was affected due to irradiation and the values measured are a reflection of this side effect. The question arises whether the extent of damage to the skin could be deduced from the determination of these values in individual cases. Thus, before a salvage operation, the risk of a wound healing disorder could be estimated and discussed with the patient. Also, prophylactic measures could be taken.

In summary, there are unfavorable conditions in terms of pH and oxygen saturation in postradiogenic wound healing disorders. However, hypoxia appears to be more pronounced and thus potentially more influential in the course of healing. In addition, we were able to objectively measure the existing damage to irradiated skin by means of 2D luminescense imaging. Whether a prognosis of wound healing can be derived from this or whether prophylactic measures can reduce the development of postradiogenic wound healing disorders should be shown in further work.

## Data Availability

The datasets generated and/or analysed during the current study are available from Steffen Auerswald (first author) and Frank Haubner (senior authtor).
